# Supplementary material for: Controlled Formation of a Protein Corona Composed of Denatured BSA on Upconversion Nanoparticles Improves Their Colloidal Stability
Source: Materials (Basel). 2021 Mar 28;14(7):1657. doi: 10.3390/ma14071657 (PMC8037850; doi:10.3390/ma14071657)
Supplement: Supplementary file 1 [file materials-14-01657-s001.pdf]

materials

Supplementary Materials

Controlled Formation of a Protein Corona Composed of Denatured BSA on Upconversion Nanoparticles Improves Their Colloidal Stability

Samah Shanwar [1], Liuen Liang [2], Andrey V. Nechaev [3], Daria K. Bausheva [1], Irina V. Balalaeva [1], Vladimir A. Vodeneev [1], Indrajit Roy [4], Andrei V. Zvyagin [1,2,5] and Evgenii L. Guryev [1,*]

1 Institute of Biology and Biomedicine, Lobachevsky State University of Nizhny Novgorod, 603950 Nizhny Novgorod, Russia; samahshanwar@gmail.com (S.S.); bausheva16@mail.ru (D.K.B.); irin-b@mail.ru (I.V.B.); v.vodeneev@mail.ru (V.A.V.); andrei.zvyagin@mq.edu.au (A.V.Z.)
2 ARC Centre of Excellence "Nanoscale BioPhotonics", Department of Physics and Astronomy, Macquarie University, Sydney 2109, Australia; liuen.liang@mq.edu.au
3 Department of Chemistry and Technology of Biologically Active Compounds, Medical and Organic Chemistry, M.V. Lomonosov Institute of Fine Chemical Technologies, MIREA-Russian Technological University, 119571 Moscow, Russia; chemorg@mail.ru
4 Department of Chemistry, University of Delhi, Delhi 110007, India; iroy@chemistry.du.ac.in
5 The Institute of Molecular Medicine, I.M. Sechenov First Moscow State Medical University, 119991 Moscow, Russia
* Correspondence: eguryev@ibbm.unn.ru

Citation: Shanwar, S.; Liang, L.; Nechaev, A.V.; Bausheva, D.K.; Balalaeva, I.V.; Vodeneev, V.A.; Roy, I.; Zvyagin, A.V.; Guryev, E.L. Controlled Formation of a Protein Corona Composed of Denatured BSA on Upconversion Nanoparticles Improves Their Colloidal Stability. *Materials* **2021**, *14*, 1657. https://doi.org/10.3390/ma14071657

Academic Editor: Jinheung Kim

Received: 4 February 2021
Accepted: 22 March 2021
Published: 28 March 2021

Publisher's Note: MDPI stays neutral with regard to jurisdictional claims in published maps and institutional affiliations.

Copyright: © 2021 by the authors. Licensee MDPI, Basel, Switzerland. This article is an open access article distributed under the terms and conditions of the Creative Commons Attribution (CC BY) license (http://creativecommons.org/licenses/by/4.0/).

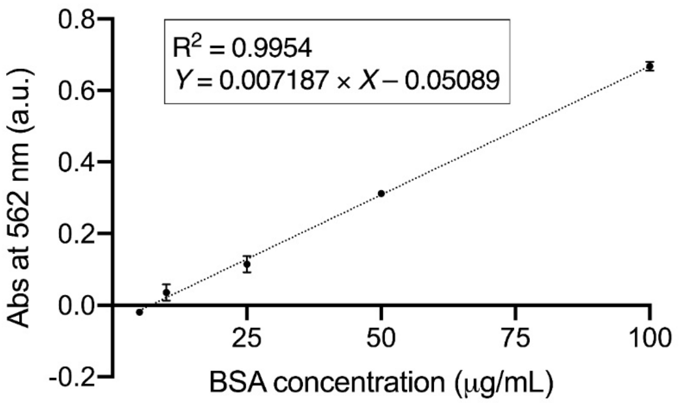

Figure S1. Calibration curve for the Pierce Micro BCA™ Protein Assay Kit.

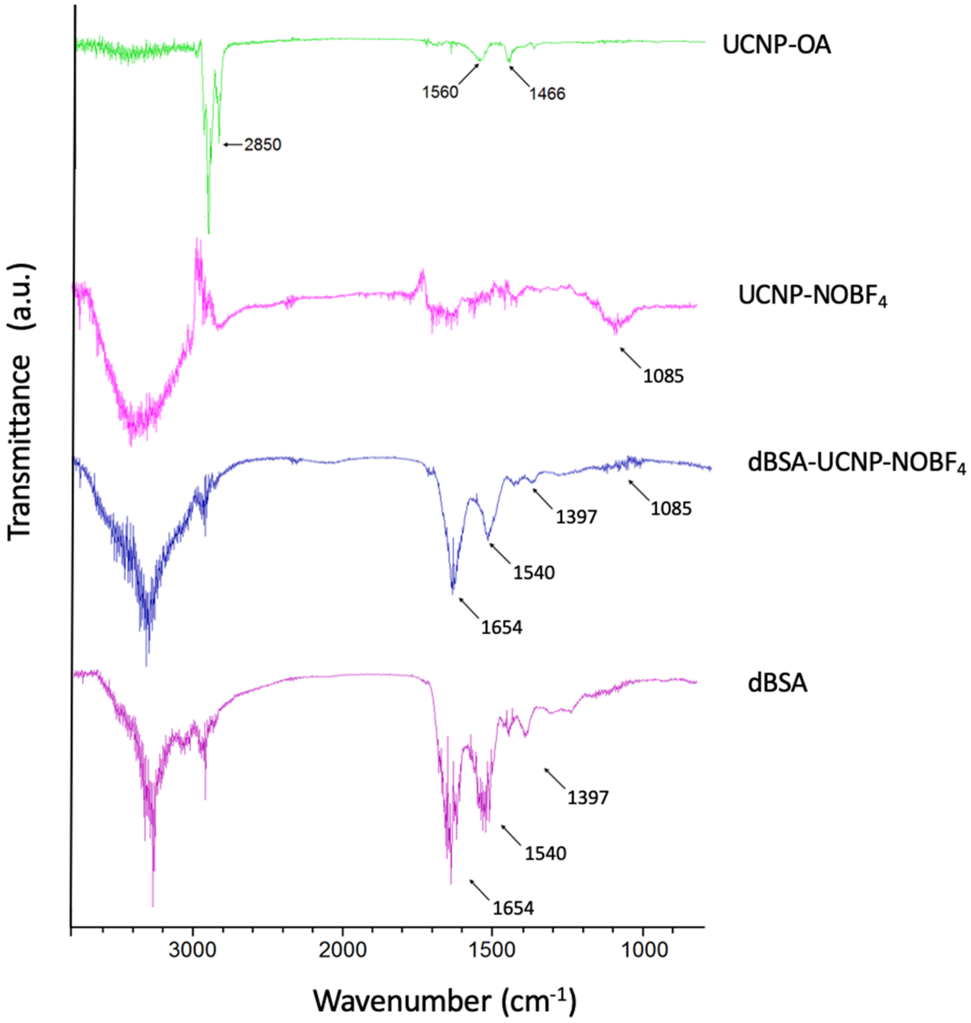

Figure S2. FTIR absorption spectra of UCNP-OA, UCNP-$NOBF_4$, and lyophilized dBSA-UCNP-$NOBF_4$; UCNP: Upconversion nanoparticles; OA: Oleic acid.

The appearance of the 2933 cm^{-1} and 2850 cm^{-1} peaks corresponds to the asymmetric and symmetric stretching vibrations of $–CH_2$ groups of oleic acid, respectively. Additionally, two bands at 1560 and 1466 cm^{-1} are observed and assigned to the asymmetric and symmetric stretch of the COO^- of oleic acid, respectively. Moreover, the $NOBF_4$ treatment of UCNP caused an intensity reduction of the peaks at 2933 and 2850 cm^{-1} and the appearance of a new band at 1085 cm^{-1} associated with BF_4^- anions. The FTIR spectrum of lyophilized dBSA-UCNP-$NOBF_4$ shows a reduction in the 1085 cm^{-1} peak corresponding to the BF_4^- anions and an alteration of the band at 1397 cm^{-1} assigned to amide III of dBSA, C≡N stretching mode and N–H bending mode.

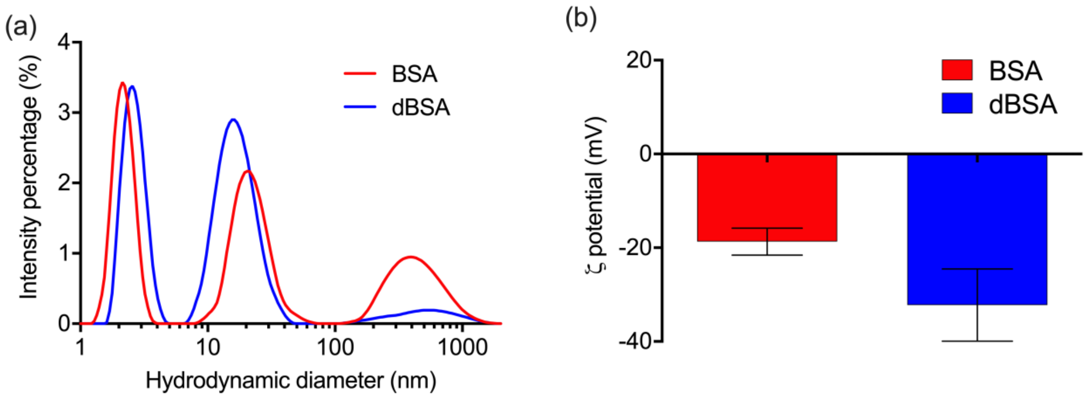

Figure S3. Characterization of bovine serum albumin (BSA) and denatured BSA by Dynamic Light Scattering and Electrophoretic Light Scattering (DLS and ELS): (**a**) hydrodynamic diameter distributions of BSA and dBSA acquired by DLS in deionized water; (**b**) the ζ-potential of BSA and dBSA acquired by ELS in deionized water.

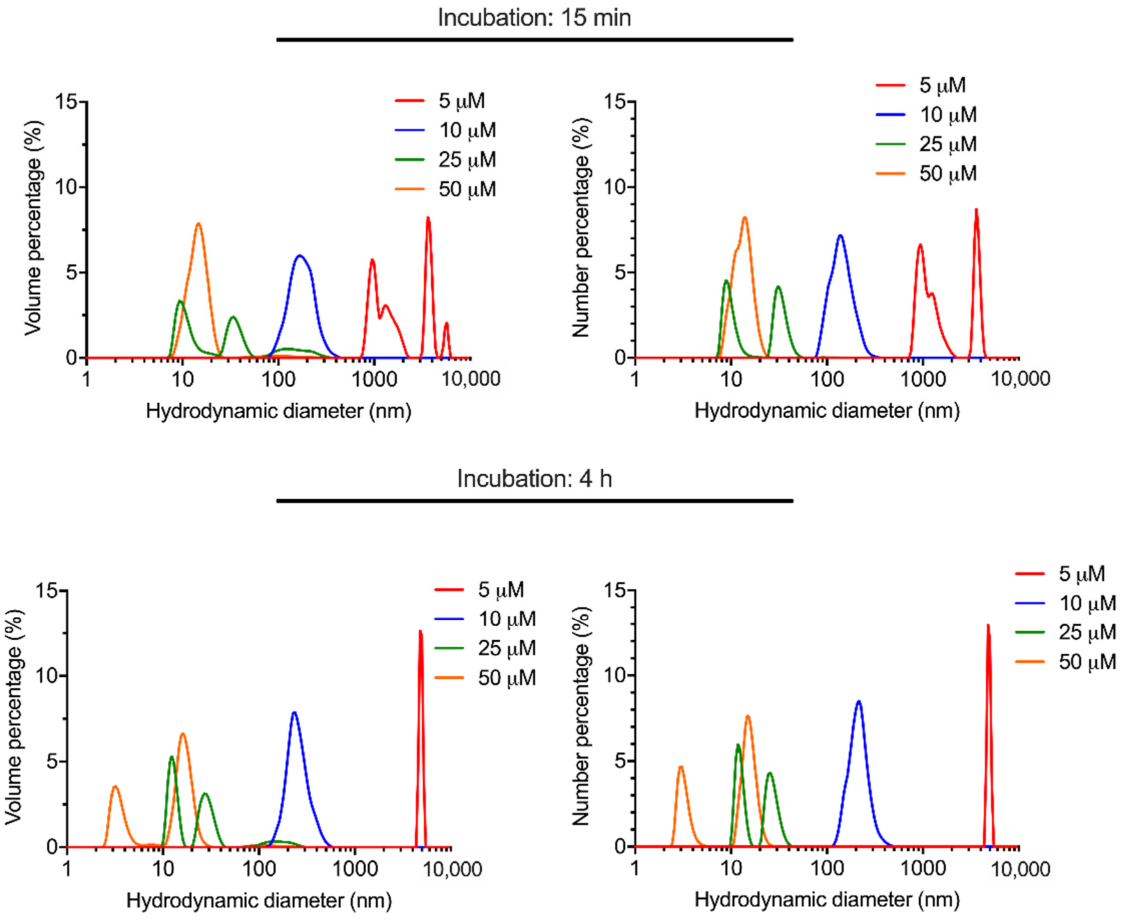

Figure S4. Concentration optimization of dBSA for forming protein corona on the surface of UCNP-$NOBF_4$ by volume and number.

Hydrodynamic diameter of dBSA-UCNP-$NOBF_4$ following the incubation of UCNP-$NOBF_4$ (0.25 mg/mL) with dBSA at different concentrations (5–50 μM) at room temperature for 15 min and 4 h measured by DLS.
